# Clinical evaluation of posterior flowable short fiber-reinforced composite restorations without proximal surface coverage

**DOI:** 10.1007/s10266-024-00905-5

**Published:** 2024-02-23

**Authors:** Rawda H. Abd ElAziz, Sherifa A. Abd ElAziz, Possy M. Abd ElAziz, Mark Frater, Pekka K. Vallittu, Lippo Lassila, Sufyan Garoushi

**Affiliations:** 1 Conservative Dentistry Department, Faculty of Dentistry, Cairo University, Cairo, Egypt; 2 Department of Operative and Esthetic Dentistry, Faculty of Dentistry, University of Szeged, Szeged, Hungary; 3 Department of Biomaterials Science and Turku Clinical Biomaterials Center–TCBC, Institute of Dentistry, University of Turku, Itäinen Pitkäkatu 4B, 20520 Turku, Finland; 4 Wellbeing Services County of South-West Finland, Turku, Finland

**Keywords:** Short fiber-reinforced composites, Randomized clinical study, EverX flow, Class II

## Abstract

The purpose of this clinical trail was to assess the clinical behavior of posterior composite restorations supported by a substantial foundation of flowable short fiber-reinforced composite SFRC (everX Flow, GC, Japan) used without proximal surface coverage with particulate filler resin composite (PFC). Seventy patients (20 males, 50 females; mean age: 30 ± 10 years) were randomly enrolled in this trial. Patients received direct restorations of either SFRC covered only on the occlusal surface (1–2 mm) by conventional PFC composite (G-ænial Posterior, GC), or plain conventional PFC composite without fiber-reinforcement, in Class II cavities in premolar and molar vital teeth. One operator made all restorations using one-step, self-etch bonding agent (G-ænial Bond, GC) according to manufacturers’ recommendations. Two blinded trained operators evaluated the restorations at baseline, at 6, 12 and 18 months using modified USPHS criteria. Results indicated that, in both groups and at different follow-up intervals, according to evaluated criteria, restorations were rated mostly with best score (Alpha) (*p* > 0.05). For the marginal integrity after 6 months, a single case in the intervention [increased to 3 (8.8%) after 18 months] and 3 (9.7%) cases of the control group [increased to 4 (12.9%) after 18 months] had Bravo score but with no significant difference (*p* > 0.05). For color match measured after 6 and 18 months, three (8.8%) cases had Bravo score in the intervention group. The use of flowable SFRC composite without any PFC surface coverage proximally in Class II restorations demonstrated satisfactory clinical outcome throughout the 18-month follow-up.

## Introduction

Nowadays the direct use of conventional particulate filler composite (PFC) is a common conservative and economic approach to restore missing tooth structures. They benefit from having a natural shade, cost less than indirect cast gold and ceramic restorations, and can adhere to enamel and dentin through bonding procedures [1]. Patient and clinician demands for natural esthetics have increased, which has resulted in the increased use of composites even in posterior teeth, where significant loading challenges arise during function [2–5]. Their application has expanded to include extra-coronal restorations as well as posterior intra-coronal restorations [2, 4].

To withstand mechanical challenges during function, modification of filler particle morphology and size led to enhanced mechanical properties [1, 6]. However, the literature shows that modern PFC composites still have drawbacks when utilized in large restorations because of their brittleness and lack of toughness [3, 7]. Owing to these limitations, there is ongoing debate regarding applying PFC composites in significant high load-bearing situations, such as direct posterior restorations in weakened coronal cavities or core build-ups [8]. The demand to enhance restorative composite has resulted in growing interest in strengthening methods. Various methods have been proposed to maintain the structural integrity of residual enamel and dentin and enhance the lifespan of large composite restorations [9]. Among them one option is using discontinuous or short flowable fiber-reinforced composites (SFRCs) to replace dentin and conventional PFC composite to replace enamel, known as a biomimetic restorative approach [10, 11]. High fracture toughness restorative materials are in high demand because they are less likely to fracture and be at risk for crack propagation. Many authors have stated that flowable SFRC (everX Flow, GC, Japan) has substantially improved mechanical features, especially in the context of fracture toughness compared to PFC composites [12–16]. According to the literature, the reinforcing capability of SFRC increased with increasing the volume of the material. Some researchers have even extended the application of SFRC beyond dentin restoration, encompassing the reconstruction of missing interproximal walls [17, 18]. This approach aims to deliver a more robust solution for the reconstructed interproximal wall when compared to conventional PFC. It is noteworthy that the manufacturer's guidelines recommend utilizing flowable SFRC primarily as a bulk base or core foundation, discouraging its use as a top surface layer.

The question arises as to why flowable SFRC (everX Flow) needs to be covered with another layer of PFC composite, even on proximal surfaces. This is despite the existence of numerous laboratory results demonstrating the favorable surface and wear characteristics of everX Flow in comparison to many commercial PFC composites [12, 19–22]. The in vitro data indicate that the use of flowable SFRC can be considered safe when exposed to the oral environment. However, there is a lack of clinical evidence to substantiate this suggestion. As a result, the goal of the current clinical trial was to investigate the clinical efficacy of posterior composite restorations reinforced by a bulk layer of flowable SFRC without proximal surface coverage. The tested null hypothesis was that SFRC in building proximal walls of Class II restorations would perform similarly to PFC restorations in terms of clinical outcomes.

## Materials and methods

### Trial design and settings

The trial was designed as a double-blinded study, involving both assessors and patients, and was conducted as a randomized control clinical study with two parallel simultaneous groups, each with an equal allocation ratio. The trail protocol was officially reported on ClinicalTrials.gov under the ID number NCT04720638. The trial took place at the Conservative Dentistry outpatient clinic of the Faculty of Dentistry at Cairo University, Egypt, spanning from March 2021 to December 2022. The reporting of this trial followed the guidelines established by the Consolidated Standards of Reporting Trials (CONSORT). Furthermore, this trial received approval from the Ethics Committee of the Faculty of Dentistry at Cairo University, with an ID number of (13/11/20). All participants were instructed about the trial's objectives and procedures, and their participation was confirmed through informed consent, which they provided by signing the forms.

### Eligibility criteria

Patients included in this study were 18–55 years old with good oral hygiene and presenting with at least one upper or lower vital posterior tooth with compound proximal carious cavity. The selected tooth should be in favorable occlusion and normal contact with the adjacent teeth. The exclusion criteria were patients with compromised medical health, pregnant women, known allergy to any material used in the trial, signs and symptoms of irreversible pulpitis or necrosis or any other pulp pathologies and previous restoration or severe periodontal problems in the selected tooth.

### Sample size calculation

Based on the findings from a prior clinical trail [23], a power analysis was devised to facilitate the application of a two-sided statistical test aimed at assessing the null hypothesis. By setting the Alpha (*α*) level at 0.05 (5%) and the beta (*β*) level at 0.20 (20%), thus ensuring a statistical power of 80%, and by determining an effect size (W) of 0.400, the projected sample size (*n*) was computed to encompass a total of 60 cases. In anticipation of potential dropouts, this number was subsequently increased by 15%, resulting in a final total of 70 cases, which corresponds to 35 cases per group. The calculations for sample size were conducted utilizing G*Power version 3.1.9.4 (citation may be needed https://link.springer.com/article/10.3758/BF03193146), a widely recognized software tool for statistical analysis.

### Randomization and concealment of allocation

The process of randomization was executed utilizing a straightforward approach overseen by a contributor who maintained no further involvement in subsequent trial phases. A total of seventy random numbers were generated employing the Random Sequence Generator (https://www.random.org/). This list of random numbers was then sealed securely within an opaque envelope. Access to this list was restricted exclusively to the operator, who could only open the envelope at the precise moment of applying the composite filling material, subsequent to the completion of the adhesive protocol. Both the two assessors responsible for evaluating trial outcomes and the patients remained unaware of the specific restorative material employed, ensuring a double-blind setup. Blinding of the operator was not applicable due to variances in the techniques of application required for the different materials.

### Interventions: restorative treatment

A single trained operator performed all the restorations. All patients received local anesthesia (Artinibsa 4% 1:100.000, Inibsa Dental, Spain). Multiple teeth isolation was done using a rubber dam (Sanctuary^®^ powder free latex dental dam, Malaysia). Preparation of all cavities was applied using # 245 and # 1, 2 round carbide burs with an air/water-cooled high speed handpiece with the aid of a sharp excavator in accordance with the cavity preparation principles for adhesive composite restorations and the recent clinical recommendations for caries excavation.

**Fig. 1:**
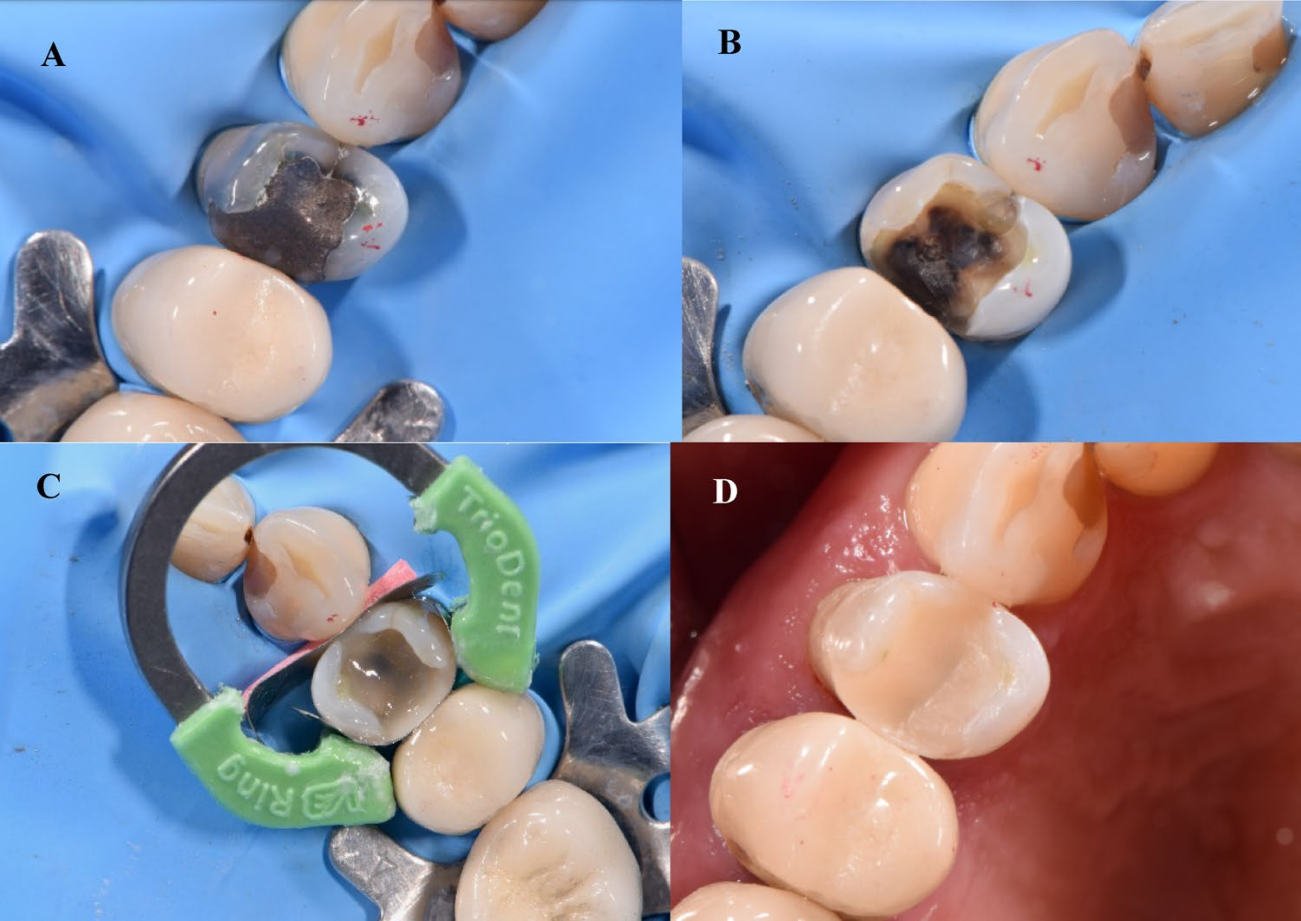
(**A**) Preoperative view; (**B**) after removal of the old restoration; (**C**) interproximal walls were built by flowable SFRC; (**D**) post-operative view

For the restorative procedures, first, the lost proximal wall was replaced using an appropriate pre-contoured sectional matrix complemented by the corresponding separating ring (TOR VM, Russia) and an appropriately dimensioned wooden wedge (Fig. 1). The prepared cavity was cleansed by applying a thorough water rinse then application of the bonding protocol. All teeth in both groups received the same adhesive protocol. A selective enamel etching approach was made by applying 37% phosphoric acid gel (Scotchbond, 3M ESPE, USA) for a duration of 15 s. Subsequently, the tooth underwent a 15 s rinsing procedure with water and was gently dried using brief air blasts and cotton pellet blotting. A one-step self-etch bonding agent application (G-ænial Bond, GC) involved a thorough and careful process where a disposable microbrush was used to gently spread the bonding agent over the surfaces of the prepared cavity. Following this application, it was left for a period of 10 s before being exposed to the highest air pressure for 5 s. Subsequently, the light-curing procedure was performed utilizing an LED light-curing unit with an intensity surpassing 700 mW/cm^2^ (LED.F, Woodpecker, China) for a total time span of 10 s.

In the intervention group, flowable SFRC (everX Flow) was injected as dentin replacement including the proximal wall (Fig. 1) leaving about 1–2 mm of space for an occlusal surface layer of the conventional PFC. SFRC was subjected to light curing for 20 s. A capping or surface layer of the conventional micro-hybrid PFC (G-ænial Posterior, GC) was applied and underwent light curing for a period of 20 s as well.

While for the control group, the cavities were completely filled with conventional PFC, using an oblique 2 mm incremental layering technique, and each increment subjected to light curing separately for 20 s.

After checking centric and eccentric occlusion by an articulating paper, restoration finishing was done, using fine grit bud-shaped diamond stones (Microdont, Brazil) then polishing by rubber points and bristle brushes with ultrafine Microdont diamond paste (Microdont). All the used materials and their specification are mentioned in Table 1.

**Table 1:** Material descriptions and specifications

Product	Specifications	Composition	
G-ænial Posterior (A2&A3)	Micro-hybrid conventional composite (PFC)	Resin Matrix: UDMA and other dimethacrylates co-monomers Filler: Pre-polymerized silica fillers, pre-polymerized silica and strontium fluoride containing fillers 81 wt%	
EverX Flow (Bulk and Dentin)	Short fiber-reinforced composite (SFRC)	Bis-EMA, TEGDMA, UDMA, micrometer scale glass fiber filler, Barium glass 70 wt%	
G-ænial Bond	One-component self-etching light-cured adhesive	HEMA-free, 4-MET, UDMA, TEGDMA, phosphoric acid monomer, acetone, water, silanated colloidal silica, initiator	

*UDMA*, urethane dimethacrylate, *TEGDMA*, triethylene glycol dimethacrylate, *Bis-EMA* Ethoxylated bisphenol-A-dimethacrylate, *HEMA* 2 hydroxyethyl methacrylate, *4-MET* 4-methacryloyloxyethyl trimellitate, *wt%* weight percentage

### Clinical evaluation

Restoration evaluation was performed by two blinded outcome investigators using modified USPHS criteria (Table 2). The examiners were trained on the modified USPHS criteria before starting the trial and calibrated to at least a kappa value of 90% per each criteria for inter- and intra-examiner agreement. The assessments of the restorations were conducted at baseline, 6-month, 12-month, and 18-month intervals. Any discrepancies in the scores were resolved through discussion.

**Table 2:** Modified USPHS criteria

Criterion	Score	Description	Measuring method
Post-operative Hypersensitivity	Alpha	Absent	Controlled air blasting at a 2 cm distance from the restoration
	Charlie	Present	
Secondary caries	Alpha	No caries present along the margins	Visual inspection with mirror
	Charlie	There is visual evidence of dark carious discoloration along the restoration	
Gross fracture	Alpha	Restoration is intact and fully retained	Visual inspection with mirror
	Bravo	some portion of the restoration is still intact and can be repaired	
	Charlie	Restoration is completely fractured	
Color match	Alpha	The restoration matches the shade and translucency of the adjacent tooth	Visual inspection with mirror
	Bravo	There is a mismatch in the shade and translucency, but it is within the normal range of tooth shade	
	Charlie	The mismatch is beyond the normal range of the tooth shades and translucency	
Cavo-surface marginal discoloration	Alpha	There is no visual evidence of any marginal discoloration at the junction of the restoration and the adjacent tooth structure	Visual inspection with mirror
	Bravo	There is visual evidence of shallow marginal discoloration	
	Charlie	There is visual evidence of deep marginal discoloration toward a pulpal direction	
Marginal integrity	Alpha	The explorer does not catch and there is no visible crevice along the margin of the restoration	Visual inspection with mirror and explorer
	Bravo	The explorer catches and there is visible evidence of a crevice but the dentin or the base are not exposed	
	Charlie	There is crevice defect extended to the dentin	
Anatomic contour (wear)	Alpha	The restoration is continued with the existing anatomic form or slightly flattened	Visual inspection with mirror and explorer
	Bravo	A surface concavity is present. But the dentin or the base is not exposed	
	Charlie	A surface concavity is present and the base and/or the dentin is exposed	
Surface texture	Alpha	Surface texture is similar to the adjacent enamel	Explorer
	Bravo	Surface texture is rougher than the adjacent enamel	
Proximal contact	Alpha	Resistance met when passing floss	Dental floss
	Bravo	Floss passed without resistance but contact present	
	Charlie	No contact with adjacent tooth	

### Statistical analysis

Numerical (age) data were expressed as mean values along with standard deviations (SD) and assessed for normality through the Shapiro–Wilk test, which confirmed their adherence to a normal distribution. Subsequently, independent *t* tests were employed for comparisons. Categorical and ordinal data were presented as percentages and frequencies. Chi-square tests were utilized for the analysis of categorical (gender and treated teeth) data, such as gender and treated teeth. For ordinal data, specifically USPHS scores, intergroup comparisons were conducted using the Mann–Whitney *U* test, while intragroup comparisons employed the Friedman’s test, followed by the Nemenyi post hoc test. A significance threshold of *p* < 0.05 was applied to all tests. The statistical analysis was executed using R statistical analysis software, version 4.1.3.

## Results

**Fig. 2:**
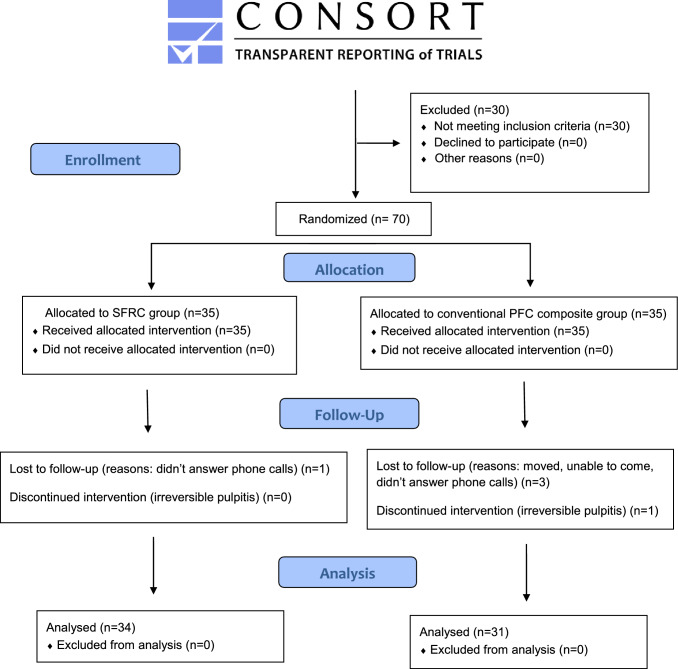
Flowchart of the study

This trail was carried on seventy cases that were randomly and equally assigned to each of the tested groups (i.e., 35 cases each). Thirty-four patients in the intervention group completed the trial while for the control group only thirty-one completed. This loss in follow-up was due to patients not replying to phone calls and one case discontinued the trial due to development of irreversible pulpitis (Fig. 2).

There were 13 (37.1%) males in the intervention group and 22 (62.9%) females, while in the control group, there were seven (20.0%) males and 28 (80.0%) females. The mean age of the cases in the intervention group was (31.51 ± 12.65) years, and in the control group, it was (29.63 ± 9.00) years. A majority of the treated teeth in the intervention group were first molars 15 (42.9%), while in the control group, they were mostly second premolars 14 (40.0%). There were no notable distinctions between the two groups in terms of various demographic data and baseline characteristics (*p* > 0.05) (Table 3).

**Table 3:** Inter-group comparison of demographic data and baseline characteristics

Parameter	Intervention	Control	*p*-value
Gender			
Male			
* n*	13	7	0.112
%	37.1%	20.0%	
Female			
* n*	22	28	
%	62.9%	80.0%	
Age (years)			
Mean ± SD	31.51 ± 12.65	29.63 ± 9.00	0.499
Treated tooth			
First premolar			
*N*	6	10	0.323
%	17.1%	28.6%	
Second premolar			
* N*	12	14	
%	34.3%	40.0%	
First molar			
*N*	15	8	
%	42.9%	22.9%	
Second molar			
* N*	2	3	
%	5.7%	8.6%	

For the marginal integrity after 6 months, a single case in the intervention (increased to 3 (8.8%) after 18 months) and three (9.7%) cases of the control group (increased to 4 (12.9%) after 18 months) had Bravo score. At all intervals, there was no significant difference between both groups (*p* > 0.05). For the intragroup comparison, there was a significant difference between scores measured at different intervals in the control group with baseline values being significantly different from values measured after 18 months (*p* = 0.039). For other parameters, there was no significant difference between different intervals (*p* > 0.05).

**Fig. 3:**
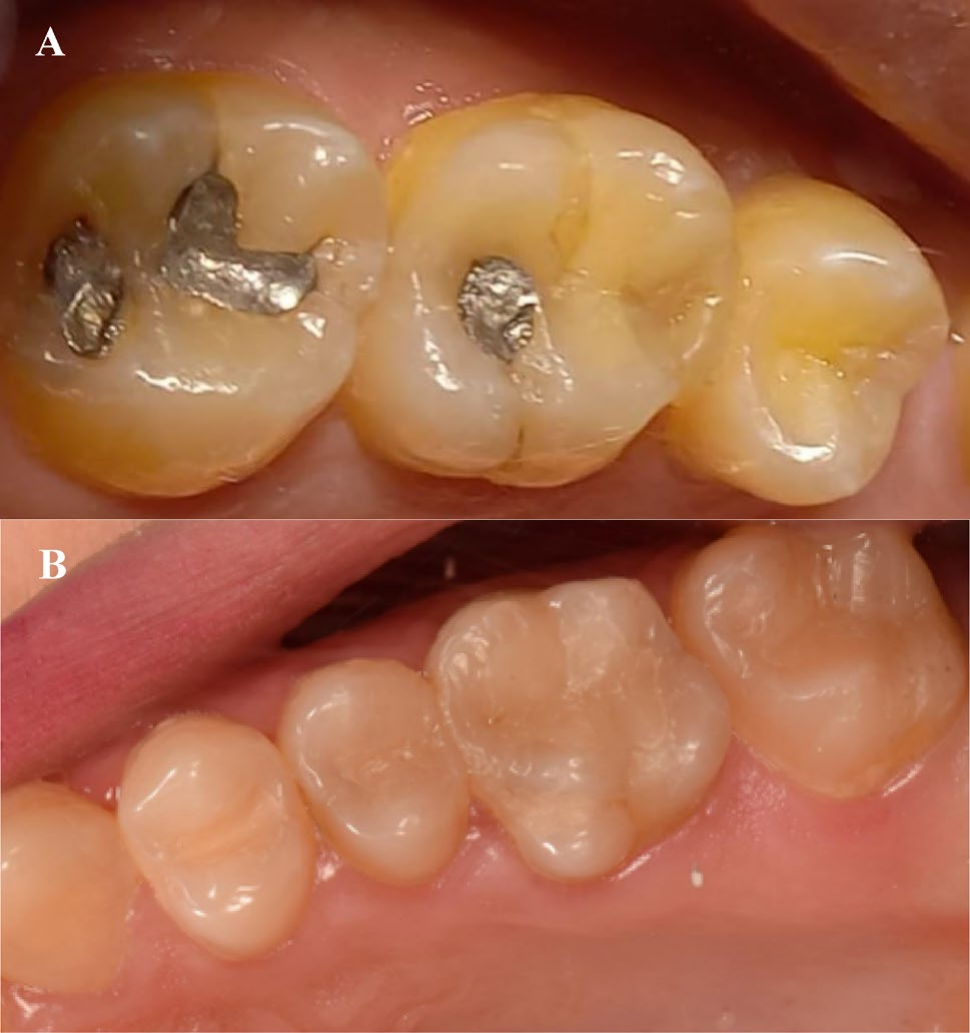
Intervention SFRC (**A**; Bravo score in color match) and control conventional PFC (**B**; alpha-score in color match) restorations in the mesial of upper first molar after 18 months

For color match measured after 6 and 18 months, three (8.8%) cases had Bravo score in the intervention group (Fig. 3), while regarding marginal discoloration, a single case in the intervention had Bravo score after 18 months.

When post-operative hypersensitivity, gross fractures, secondary caries, proximal contact, anatomical contour (wear), and surface texture were assessed, every case in both groups observed at various follow-up intervals received an Alpha score (Table 4). It is also important to highlight that there were no signs of gingival irritation or inflammation around the restorations in both the control and intervention groups.

**Table 4:** Inter- and intragroup comparisons of modified USPHS criteria

Parameter	Score	Baseline				6 months				18 months				*p*-value	
		Intervention		Control		Intervention		Control		Intervention		Control			
		*n*	%	*n*	%	*n*	%	*n*	%	*n*	%	*n*	%	Intervention	Control
Post-operative hypersensitivity/secondary caries	Alpha	35	100.0%	35	100.0%	34	100.0%	31	100.0%	34	100.0%	31	100.0%	NA	NA
	Charlie	0	0.0%	0	0.0%	0	0.0%	0	0.0%	0	0.0%	0	0.0%		
*p*-value		NA				NA				NA					
Gross fracture/proximal contact/anatomic contour (wear)/surface texture	Alpha	35	100.0%	35	100.0%	34	100.0%	31	100.0%	34	100.0%	31	100.0%	NA	NA
	Bravo	0	0.0%	0	0.0%	0	0.0%	0	0.0%	0	0.0%	0	0.0%		
	Charlie	0	0.0%	0	0.0%	0	0.0%	0	0.0%	0	0.0%	0	0.0%		
*p*-value		NA				NA				NA					
Color match	Alpha	35	100.0%	35	100.0%	31	91.2%	31	100.0%	31	91.2%	31	100.0%	0.051	NA
	Bravo	0	0.0%	0	0.0%	3	8.8%	0	0.0%	3	8.8%	0	0.0%		
	Charlie	0	0.0%	0	0.0%	0	0.0%	0	0.0%	0	0.0%	0	0.0%		
*p*-value		NA				0.096				0.096					
Cavo-surface marginal discoloration	Alpha	35	100.0%	35	100.0%	34	100.0%	31	100.0%	33	97.1%	31	100.0%	0.368	NA
	Bravo	0	0.0%	0	0.0%	0	0.0%	0	0.0%	1	2.9%	0	0.0%		
	Charlie	0	0.0%	0	0.0%	0	0.0%	0	0.0%	0	0.0%	0	0.0%		
*p*-value		NA				NA				0.355					
Marginal integrity	Alpha	35	100.0%	35^A^	100.0%	33	97.1%	28^AB^	90.3%	31	91.2%	27^B^	87.1%	0.097	0.039*
	Bravo	0	0.0%	0	0.0%	1	2.9%	3	9.7%	3	8.8%	4	12.9%		
	Charlie	0	0.0%	0	0.0%	0	0.0%	0	0.0%	0	0.0%	0	0.0%		
*p*-value		NA				0.269				0.608					

Values with different superscript letters within the same horizontal row and group are significantly different *significant (*p* < 0.05), *NA* Not applicable

## Discussion

One of the recent innovations in restorative dentistry is the introduction of flowable SFRC composite (everX Flow) for reinforcing posterior teeth. Comparative research in the literature has demonstrated that everX Flow exhibits unique characteristics, composition, and strengthening capabilities when compared to both conventional and SFRC composites [15, 24].

While numerous laboratory studies have been carried out to assess the surface characteristics of this recently presented restorative material, their results may not consistently replicate real clinical behavior. Hence, conducting clinical studies remains the most reliable method for predicting its actual performance when exposed to the oral environment. Only two short-term clinical studies involving the application of everX Flow have been published recently [25, 26]. In these studies, the material was compared either to packable SFRC (everX Posterior, GC Europe) or to glass hybrid (Equia Forte^®^ HT, GC Europe) materials. The clinical performance of everX Flow restorations proved to be comparable with packable SFRC, and more successful than glass hybrid restorations in terms of retention. However, everX Flow was used as a dentin replacing material and was not exposed to the oral environment. As far as we are aware, this is the first clinical trail investigating the clinical performance of flowable SFRC reinforced Class II direct restorations where interproximal walls were built using SFRC and without PFC surface coverage. Therefore, the clinical success of SFRC Class II composite restorations cannot be compared with the literature. The tested restorative approach performed similarly to conventional PFC restorations in all parameters throughout the course of the 18-month testing time frame, thereby confirming the hypothesis.

EverX Flow was introduced in 2019 as the flowable version of packable SFRC (everX Posterior) manufactured by the same company (GC, Japan). It is made up of an inorganic silanated particle filler (45 wt%), a resin matrix (30 wt%), and randomly oriented glass microfibers (25 wt%). The diameter of the used glass microfibers is 6 µm and the length between 200 and 300 µm [20]. By comparison, everX Posterior has fiber of 17 μm in diameter and length in the range of 0.3–1.5 mm [15, 20]. Hence, it is unsurprising that everX Flow exhibits superior surface properties when compared to everX Posterior [15]. However, the official manufacturer’s instruction remains to cover everX Flow always from proximal and occlusal surfaces with a layer of conventional PFC composite.

When compared to other conventional and bulk-fill PFCs, laboratory studies on flowable SFRC revealed good wear resistance characteristics [20, 21, 27]. Within the mentioned research, the microfibers of the flowable SFRC did not protrude; instead, they were polished in the resin matrix, resulting in often a uniformly smooth surface. In another studies by Lassila et al., and Uctasli et al., flowable SFRC showed surface gloss values and color stability which was comparable to other tested conventional PFC and fluoride-releasing composites [19, 22]. These studies reported that polished surfaces of flowable SFRC was smooth compared to those where PFC composites were used. In accordance with these laboratory findings, all SFRC and PFC restorations assessed in this clinical trial scored Alpha for proximal contact, anatomic contour (wear) and surface texture (Table 4).

In the present study, by the conclusion of the 18-month monitoring time, three SFRC restorations (8.8%) had Bravo score (clinically acceptable) for color match, while regarding marginal discoloration, a single case in the intervention had also Bravo score. This outcome can be attributed to the use of the bulk shade translucent color of everX Flow, since the residual layer of the surface PFC composite (G-ænial Posterior) was thin after occlusal adjustment. Subsequently, a thin layer of PFC proved insufficient to mask the dark color of any potential leftover amalgam tattoo or dark sclerotic dentin (Fig. 2).

The degree of monomer conversion (DC%), the level of water sorption, and the hydrophilicity of the resin matrix can all have an impact on a composite's discoloration resistance [22, 28]. A higher DC% indicates a lower amount of unreacted monomers, reduced water uptake, and enhanced color stability. Based on the available literature [20, 29], it has been reported that G-ænial Posterior has a lower DC% (48) compared to everX Flow (63). This difference in DC% may account for the good color stability of SFRC. It is thought that water acts as a conduit for stains to enter the resin matrix [30]. The particulate fillers or fibers incorporated within the composites do not exhibit water absorption into the material's core; however, they can absorb water at their surface. In addition, the shade of the composite is another aspect influencing composite staining. Darker shades (dentin shade) generally exhibit improved color matching capabilities owing to the existence of pigments.

From our results, though significant difference was not detected, three SFRC and four PFC restorations scored Bravo for marginal integrity after the follow-up period. It has been claimed that polymerization shrinkage and stress represent the principal contributors to insufficient marginal adaptation. In fact, comparing monomers used in PFC and SFRC composites, TEGDMA, which has low molecular weight and viscosity, can produce high shrinkage stresses [31]. This effect is due to the increased reactivity of this monomer, which causes high conversion and therefore high shrinkage. Nevertheless, recent studies in the literature have indicated that utilizing SFRC for restoring large cavities results in fewer polymerization shrinkage-related cracks compared to layered conventional PFC composites [14, 32]. This observation appears to have a direct clinical significance.

It is also worth noting that Lassila et al., in their investigation of Streptococcus mutans adhesion, demonstrated that the SFRC material exhibited a level of Streptococcus mutans adhesion similar to that of commercial PFC composites [33]. Furthermore, Attik et al. demonstrated that everX Flow had a less detrimental effect on the viability of primary gingival cells compared to other tested bulk-fill PFC composites [12]. Consistently, none of the restorations in this study exhibited specific signs of soft tissue irritation or plaque accumulation.

Although the manufacturer recommends using everX Flow exclusively to substitute missing dentin for direct and indirect restorations, and avoiding exposure to the oral environment, the study’s findings suggest that flowable SFRC could potentially be utilized safely across a wider range of applications. However, it is necessary to conduct clinical trails with larger sample sizes and extended follow-up to obtain more reliable and conclusive findings.

## Conclusion

The application of flowable short fiber-reinforced composite, without proximal surface coverage by conventional micro-hybrid composite in Class II restorations, yielded satisfactory clinical outcomes throughout the 18-month follow-up, as assessed by the modified USPHS criteria.

## Data Availability

Data available within the article.
